# Complete genome sequence and phenotype microarray analysis of *Cronobacter sakazakii* SP291: a persistent isolate cultured from a powdered infant formula production facility

**DOI:** 10.3389/fmicb.2013.00256

**Published:** 2013-09-02

**Authors:** Qiongqiong Yan, Karen A. Power, Shane Cooney, Edward Fox, Gopal R. Gopinath, Christopher J. Grim, Ben D. Tall, Matthew P. McCusker, Séamus Fanning

**Affiliations:** ^1^UCD Centre for Food Safety, WHO Collaborating Centre for Research, Reference and Training on Cronobacter, School of Public Health, Physiotherapy and Population Science, University College DublinDublin, Ireland; ^2^Division of Virulence Assessment, OARSA, Centre for Food Safety and Applied Nutrition, MOD 1 Facility, Virulence Mechanisms Branch (HFS-025), U.S. Food and Drug AdministrationLaurel, MD, USA

**Keywords:** complete genome, plasmid, *Cronobacter sakazakii*, stress response, antibiotic resistance, toxic compounds

## Abstract

Outbreaks of human infection linked to the powdered infant formula (PIF) food chain and associated with the bacterium *Cronobacter*, are of concern to public health. These bacteria are regarded as opportunistic pathogens linked to life-threatening infections predominantly in neonates, with an under developed immune system. Monitoring the microbiological ecology of PIF production sites is an important step in attempting to limit the risk of contamination in the finished food product. *Cronobacter* species, like other microorganisms can adapt to the production environment. These organisms are known for their desiccation tolerance, a phenotype that can aid their survival in the production site and PIF itself. In evaluating the genome data currently available for *Cronobacter* species, no sequence information has been published describing a *Cronobacter sakazakii* isolate found to persist in a PIF production facility. Here we report on the complete genome sequence of one such isolate, *Cronobacter sakazakii* SP291 along with its phenotypic characteristics. The genome of *C. sakazakii* SP291 consists of a 4.3-Mb chromosome (56.9% GC) and three plasmids, denoted as pSP291-1, [118.1-kb (57.2% GC)], pSP291-2, [52.1-kb (49.2% GC)], and pSP291-3, [4.4-kb (54.0% GC)]. When *C. sakazakii* SP291 was compared to the reference *C. sakazakii* ATCC BAA-894, which is also of PIF origin, the annotated genome data identified two interesting functional categories, comprising of genes related to the bacterial stress response and resistance to antimicrobial and toxic compounds. Using a phenotypic microarray (PM), we provided a full metabolic profile comparing *C. sakazakii* SP291 and the previously sequenced *C. sakazakii* ATCC BAA-894. These data extend our understanding of the genome of this important neonatal pathogen and provides further insights into the genotypes associated with features that can contribute to its persistence in the PIF environment.

## Introduction

*Cronobacter* species (formerly *Enterobacter sakazakii*) is an opportunistic pathogen of the Enterobacteriaceae family. This organism was originally designated as *E. sakazakii* in 1980 (Farmer et al., 1980). Based on its recently revised taxonomy, the genus was renamed as *Cronobacter* in 2007 and now consists of seven species, *C. sakazakii*, *C. malonaticus*, *C. turicensis*, *C. muytjensii*, *C. dublinensis* (including three subspecies, *dublinensis*, *lausannensis*, and *lactaridi*), *C. universalis* and *C. condimenti* (Iversen et al., 2004, 2007, 2008; Joseph et al., 2011). Infections caused by *Cronobacter* can present as necrotizing enterocolitis, bacteremia and meningitis, with long term complications for those that survive, including delayed neurological development, hydrocephalus and permanent neurological damage. Life-threatening infections have been reported in neonates (of less than 28 days) (Bar-Oz et al., 2001; Gurtler et al., 2005; Mullane et al., 2007), as well as older infants, with lethality rates ranging between 40 and 80% (Bowen and Braden, 2006; Friedemann, 2009), and more recently in immune-compromised adults, mainly the elderly (Gosney et al., 2006; See et al., 2007; Hunter et al., 2008; Tsai et al., 2013).

*Cronobacter* can be isolated from a wide range of foods and environments (Baumgartner et al., 2009; Chap et al., 2009; El-Sharoud et al., 2009; Jaradat et al., 2009; Schmid et al., 2009). Specifically, contaminated powdered infant formula (PIF) has been epidemiologically linked with many of the neonatal and infant infections (Himelright et al., 2002; Bowen and Braden, 2006; Mange et al., 2006). Previous studies reported the isolation of *Cronobacter* from PIF, and the PIF production environment (Drudy et al., 2006; Mullane et al., 2008a,b), suggesting that this bacterium has the capacity to adapt to, survive and persist under desiccated environmental conditions. Comparison of environmental and clinical *Cronobacter* isolates, indicated that the desiccation tolerance exhibited might play a role in the persistence of *Cronobacter* in PIF and its associated low-moisture ingredients (Walsh et al., 2011; Beuchat et al., 2013). Stress response factors identified previously in *Cronobacter*, which include heat-shock, cold-stresses, survival in dry conditions, water activity (a_w_), and pH may contribute to this phenotype (Dancer et al., 2009a,b; Carranza et al., 2010; Chang et al., 2010; Arku et al., 2011). Genome sequencing efforts of *Cronobacter* species commenced in 2010. To date, 16 genomes are currently available, of which three, *C. sakazakii* ATCC BAA-894, *C. sakazakii* ES15 and *C. turicensis* z3032, are complete (Kucerova et al., 2010; Stephan et al., 2011; Joseph et al., 2012; Shin et al., 2012; Grim et al., 2013).

Following on-going surveillance of a PIF production facility in our laboratory, an interesting isolate *C. sakazakii* SP291 was identified which exhibited a thermo-adapted phenotype when compared with other *Cronobacter* and *Salmonella* species tested under laboratory conditions (Cooney, 2012). In an effort to better understand *C. sakazakii* SP291, its genome was completely sequenced and compared to that of a PIF isolate *C. sakazakii* ATCC BAA-894, a whole grain isolate *C. sakazakii* ES15, a clinical isolate *C. turicensis* z3032 and other selected draft genomes. Additionally, we interrogated the phenome of *C. sakazakii* SP291, to determine the functionality of strain-specific genotypic traits that may contribute to its adaption capacity in a PIF production environment.

## Materials and methods

### Bacterial isolates studied and their culture conditions

Seventeen *Cronobacter* isolates used in this study are listed in Table 1. *Cronobacter sakazakii* SP291 was assigned according to the classic *rpo*B method described previously (Stoop et al., 2009; Lehner et al., 2012). The isolate was cultured routinely in an Isotherm® Forced Convection Laboratory Incubator (Esco GB Ltd., Downton, UK) at 37°C on Trypticase Soy Agar (Oxoid Limited, Hampshire, UK) and stored at −80°C on cryo-beads (Technical Service Consultants Ltd., Lancashire, UK).

**Table 1:** ***Cronobacter* species, the strain identifier, source, country of origin, and accession numbers**.

**Species**	**Strain identifier^a^**	**Serogroup^b^**	**Source**	**Country of origin**	**Accession number (GeneBank)**
*Cronobacter sakazakii*	ATCC BAA-894	*Csak* O1	PIF^g^	USA	CP000783-CP00785
*Cronobacter sakazakii*	SP291	*Csak* O2	PIF manufacturing environment	Ireland	CP004091-CP004094
*Cronobacter sakazakii*	ES15	ND^f^	Whole grain	Korea	CP003312
*Cronobacter sakazakii*	E899	*Csak* O2	Clinical	USA	AFMO01000001-AFMO01000385
*Cronobacter sakazakii*	680	ND^f^	Clinical	USA	CALG01000001-CALG01000201
*Cronobacter sakazakii*	696	ND^f^	Clinical	France	CALF01000001-CALF01000569
*Cronobacter sakazakii*	701	ND^f^	Clinical	France	CALE01000001-CALE01000768
*Cronobacter sakazakii*	ES35	*Csak* O1	Clinical	Israel	AJLC01000001-AJLC01000183
*Cronobacter sakazakii*	2151	*Csak* O2	Clinical, cerebrospinal fluid	USA	AJKT01000001-AJKT01000060
*Cronobacter sakazakii*	ES713	*Csak* O2	PIF	USA	AJLB01000001-AJLB01000156
*Cronobacter sakazakii*	E764	*Csak* O4	Clinical	Czech Republic	AJLA01000001-AJLA01000032
*Cronobacter malonaticus*	LMG 23826	*Cmal* O2	Human, breast abscess	USA	CALC01000001-CALC01000171
*Cronobacter turicensis*	z3032^c^	*Ctur* O1	Neonate	Switzerland	FN543093-FN543096
*Cronobacter dublinensis*	CFS 237^d^	*Cdub* O1	PIF	Ireland	CAKZ01000001-CAKZ01000221
*Cronobacter mutjensii*	ATCC 51329	*Cmuy*O2	Unknown	Unknown	AJKU01000001-AJKU01000072
*Cronobacter universalis*	NCTC 9529	*Cuni* O1	Water	UK	CAKX01000001-CAKX01000231
*Cronobacter condimenti*	1330^e^	ND^f^	Spiced meat	Slovakia	CAKW01000001-CAKW01000155

a Strain information was selected from publications (Kucerova et al., 2010; Chen et al., 2011; Stephan et al., 2011; Joseph et al., 2012; Shin et al., 2012; Grim et al., 2013).

b Serogroup designations were identified using primers described by Mullane et al. (2008a,b) and Jarvis et al. (2011, 2013).

c Cronobacter turicensis species type strain LMG 23827.

d Cronobacter dublinensis species type strain LMG 23823.

e Cronobacter condimenti species type strain LMG 26250.

f Not determined.

g Isolate cultured from PIF, of which the PFGE pattern matched the blood sample of an infected neotate in a neonatal intensive care unit (NICU) in Tennessee in 2001. The infection cause the death of the neotate born 20 days previously.

### DNA sequencing, annotation, and comparative genomic analysis

Total genomic DNA was purified using a DNeasy Blood and Tissue Kit (QIAGEN, Hilden, Germany) following the manufacturer's instructions. Concentrations were measured using a Nanodrop® (ND 1000) spectrophotometer (Labtech International Ltd., Luton, UK). Purified DNA was maintained at −20°C until required. The whole genome sequencing and assembly methodology is described elsewhere (Power et al., 2013). The complete chromosome and plasmid sequences were uploaded to the RAST (Rapid Annotation using Subsystem Technology) (Aziz et al., 2008) annotation server in a FASTA file format. The RAST server automatically identifies protein-encoding, tRNA and rRNA genes, assigns their functions, predicts the presence of subsystems in the genome, and reconstructs the metabolic network (Aziz et al., 2008). Genome to genome comparative analysis was performed in the SEED viewer as previously reported (Overbeek et al., 2005; Grim et al., 2013). Three complete genomes of *C. sakazakii* ATCC BAA-894 (Accession number CP000783-CP00785), *C. sakazakii* ES15 (Accession number CP003312) and *C. turicensis* z3032 (Accession number FN543093-FN543096) were uploaded and annotated in RAST, and used as reference sequences. Most probable insertion or deletion genome regions of *C. sakazakii* SP291 were identified as previously reported (Grim et al., 2013). In addition, nitrogen metabolism genes, stress-coding genes, as well as antibiotic and toxic compound resistant genes were determined based on significant identity alignments using BLAST. The genome sequence of *C. sakazakii* SP291 was deposited in GenBank under the accession numbers CP004091-CP004094. The accession numbers for other genome sequences studied were included in Table 1.

### Phenotype microarray analysis

Phenotype microarray (PM) analysis was performed on *C. sakazakii* ATCC BAA-894 and *C. sakazakii* SP291 using the OmniLog® automated incubator/reader (Biolog Inc., Hayward, USA) following manufacturer's instruction. All 20 plates (PM-1 through PM-20) inoculated with bacterial cell suspensions, were incubated at 37°C and cell respiration was measured every 15 min for 48 h. The tetrazolium redox dye is reduced when bacteria respire, which provides both amplification and quantitation of the phenotype. Analysis was carried out using OmniLog® phenotype microarray software v1.2 to determine the phenotypic differences. Negative control wells, which contained the inoculated Omnilog™ growth medium, but without any substrate, were measured to normalize differences in inocula and redox dye oxidation between samples. The respiration profiles for both strains were compared using PM's integration function software and a significant divergent phenotype was identified when a difference in Omnilog™ units of 20,000 ± 1800 or greater between the two strains was obtained.

## Results and discussion

### *Cronobacter sakazakii* SP291 genome

**Figure A1:**
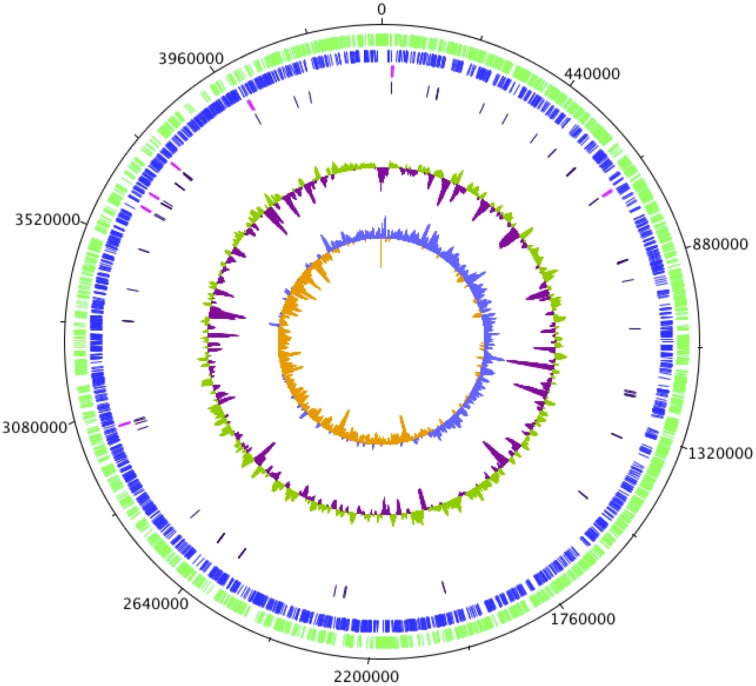
**Circular representation of the *C. sakazakii* SP291 genome**. The first circle represents the scale in kilobases starting with the origin of replication at position 0. The second circle shows the distribution of CDS (Goldman and Green, 2009) in forward strand. The third circle indicates the distribution of CDS (blue) in reverse strand. rRNA operons are colored in red on the fourth circle. tRNA operons are colored in purple on the fifth circle. The sixth circle indicates the deviation of the GC content average, with values greater than zero in green and those less than zero in purple. The innermost circle displays the GC skew ([G + C]/[G − C]), with values greater than zero in light blue and those less than zero in orange. The figure was generated using DNAPlotter (Carver et al., 2009).

The complete genome sequence of *C. sakazakii* SP291 is composed of a single, circular chromosome, 4.34 Mb in length with an average GC content of 56.9% along with three plasmids (denoted as pSP291-1, 118.136 kb, 57.2% GC; pSP291-2, 52.134 kb, 49.2% GC and pSP291-3, 4.422 kb, 54.0% GC) (Accession number CP004091-CP004094). The general features of the genome are presented in Table 2. A total of 4129 genes were identified on the chromosome, including 82 tRNA and 22 rRNA genes. The protein coding sequence (CDS) represents 86.3% of the genome and is organized into 4025 CDS, with an average length of 931 nucleic acids (Figure A1). From the annotation of the three plasmids, it was determined that 116 genes cover 87.1% of pSP291-1, 74 genes cover 77.2% of pSP291-2 and 7 genes were located on pSP291-3 and accounts for 48.6% of this structure.

**Table 2:** **General features of the *C. sakazakii* SP291 genome**.

**Feature**	**Chromosome**	**Plasmids**		
		**pSP291-1**	**pSP291-2**	**pSP291-3**
Size (bp)	4,344,092	118,136	52,134	4,422
Predicted CDS	4025	116	74	7
GC content (%)	56.9	57.2	49.2	54.0
Coding regions (%)	86.3	87.1	77.2	48.6
Average CDS length (bp)	931	887	544	307
tRNA	82	nil	nil	nil
rRNA	22	nil	nil	nil

### Comparative genomic analysis of *C. sakazakii* SP291 with three other completed *Cronobacter* genomes

**Figure 1:**
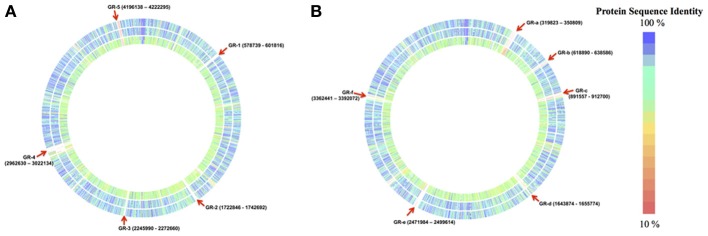
**Genomic regions of *C. sakazakii* SP291 compared to three completed genomes consists of *C. sakazakii* ATCC BAA 894, *C. sakazakii* ES15, and *C. turicensis* z3032. (A)** Regions absent in the genome of *C. sakazakii* SP291 compared to the other three genomes. Reference genome: *C. sakazakii* ATCC BAA-894; Outer circle: *C. sakazakii* ES15; Middle circle: *C. sakazakii* SP291; Inner circle: *C. turicensis* z3032, see also Table S1. **(B)** Regions present in the genome of *C. sakazakii* SP291 compared to the other three genomes. Reference genome: *C. sakazakii* SP291; Outer circle: *C. sakazakii* ATCC BAA-894; Middle circle: *C. sakazakii* ES15; Inner circle: *C. turicensis* z3032, see also Table S2.

*Cronobacter sakazakii* SP291 and three other completed genomes: *C. sakazakii* ATCC BAA-894, *C. sakazakii* ES15 and *C. turicensis* z3032 were compared (Figure 1). For the purposes of this comparison, the *C. sakazakii* ATCC BAA-894 genome was used as the reference. Five genomic regions (denoted as GR-1 through −5, in Figure 1A) were identified and these were present in the other genomes but missing in *C. sakazakii* SP291 (Table S1). These GRs are discussed in detail below.

Several unique prophages or phage-like elements of *C. sakazakii* ATCC BAA-894 were noted in GR-1 (genome positions 578,739…601,816), GR-3 (genome positions 2,245,990…2,272,660) and GR-4 (genome positions 2,962,630…3,022,134). Three specific genes were observed in GR-1, which included the DNA-methyltransferase subunit M and the S subunit of a type I restriction-modification system, along with a zinc binding domain/DNA primase, which is a phage P4-associated/replicative helicase denoted as RepA. A lambda phage portal protein, a large subunit of a terminase enzyme, along with some hypothetical proteins were noted in GR-3. In GR-4, a 1478 bp uncharacterized translocase gene required for O-antigen conversion and two-recombination genes, part of the bacteriophage *ninR* region, and denoted as *ninB* and *ninG* were identified. These annotations confirmed those previous reported (Kucerova et al., 2010). Interstingly, a putative bactoprenol glucosyl transferase was identified in *C. sakazakii* ATCC BAA-894, and shared with *C. sakazakii* ES15 and *C. turicensis* z3032, but not *C. sakazakii* SP291. Regulatory protein CII along with a phage Kil protein were annotated in *C. sakazakii* ATCC BAA-894 but not *C. sakazakii* SP291. Although protein CII was absent in *C. turicensis* z3032, the Kil protein was present (Stephan et al., 2011). A recently sequenced phage denoted as, phiES15, contained *cII* and *kil* (Lee et al., 2012). Unique transposon genes were noted in GR-2 (genome positions 1,722,846…1,742,692) and GR-5 (genome positions 4,196,138…4,222,295). In GR-2, a large part of the region containing tellurium resistance-encoding genes, including *terX, terW, terA, terB*, *terC*, and *terD*, were identified, a feature reported previously (Kucerova et al., 2010; Joseph et al., 2012; Grim et al., 2013). GR-5 contained heavy metal efflux and resistance genes, which consists of *cusS*, *cusR*, *cusC*, *cusF*, *czcB*, *czcA*, *cusA*, *copG*, *pcoS, pcoB*, and *pcoA* (Kucerova et al., 2010; Joseph et al., 2012). Further detailed information related to the corresponding phenotypes is outlined below (see also Table S1).

Genes unique to *C. sakazakii* SP291 were also noted and these were used as a reference to interrogate the genomes of the other strains. Six genomic regions (Figure 1B, denoted as GR-a through -f, Table S2) were identified as being unique to *C. sakazakii* SP291. GR-a (genome regions 319,823…350,809), GR-d (genome regions 1,643,874…165,774) and GR-e (genome regions 2,471,984…2,499,614) contained a set of phage- and phage-related proteins along with some hypothetical proteins. A phage regulatory CII-like protein was identified in *C. sakazakii* SP291 and mapped within GR-a, which also matched a similar homolog observed in *C. turicensis* z3032. A holin protein, which controls the length of an infective cycle for bacteriophage (Wang et al., 2000), together with membrane proteins related to metalloendopeptidases were present in *C. sakazakii* SP291 alone, being located in GR-e. In GR-b (genome regions 618,890…638,586), a YkfI toxin-encoding protein along a YfjZ-antitoxin encoding protein (the corresponding antitoxin to YpjF) were identified and unique to *C. sakazakii* SP291. This toxin-antitoxin protein pair was also reported in *E. coli* previously and was shown to regulate cell death through the disruption of essential cellular processes (Brown and Shaw, 2003). It has been proposed by Lewis (2000) that, under some circumstances, it may be evolutionarily advantageous for some cells in a population to undergo programmed cell death in order to provide nutrients for the remainder. Toxin-antitoxin pairs were noted in a previous study as most *Cronobacter* genomes contain a large number of them, which might be conserved, shared, or unique (Grim et al., 2013). GR-c (genome regions 891,557…912,700) contains seven interesting genes, which includes an uncharacterized protein YeeT, a NgrB protein, an ATP-dependent Clp protease, an ATP-binding subunit ClpA, a small HspC2 heat shock protein, a galactoside O-acetyltransferase-encoding gene and an anti-restriction protein KlcA which have been reported as a component part of a type I DNA restriction system (Serfiotis-Mitsa et al., 2010). A helicase protein, a glycerol dehydrogenase enzyme-encoding gene, and a DNA-cytosine methyltransferase were identified within GR-f (genome regions 3,363,441…3,392,072). Of note, a type I restriction-modification system, specificity the S-subunit-like gene, was identified in *C. sakazakii* SP291, a feature which was noted earlier in *C. sakazakii* ATCC BAA-894 (Kucerova et al., 2010; Joseph et al., 2012).

### Comparative genomic analysis of *C. sakazakii* SP291 and selected available genomes within this genus

Two earlier studies described the core genome of *Cronobacter* (Joseph et al., 2012; Grim et al., 2013). The availability of *C. sakazakii* SP291 genome has provided an opportunity to re-evaluate the content of the *Cronobacter* core gemome, comparing it to other currently available genome sequences within the genus. Thus, a comparison between *C. sakazakii* SP291 and 16 other *Cronobacer* genomes (Table 1) was performed in SEED viewer server.

Within the 11 *Cronobacter sakazakii* isolates compared, 57 annotated genes were present in *C. sakazakii* SP291, but absent in all other genomes, including 41 hypothetical proteins, 12 phage- and prophage-related genes/proteins and four other genes/proteins (Table S3). Among all seven *Cronobacter* species, there were 154 annotated genes/proteins absent in other species, including 122 hypothetical proteins, 4 phage- and prophage-related genes/proteins and 28 unique genes/proteins (Table S4). Interestingly, a conserved domain protein was identified that was unique to *C. sakazakii* SP291, which is associated with retron-type reverse transcriptase. Fifteen genes were shared with other species by *C. sakazakii* SP291, but were absent in all the *C*. *sakazakii* genomes compared to date, and these consisted of a retron-type RNA-directed DNA polymerase, a holin protein which controls the timing of bacteriophage infections as mentioned earlier, a topoisomerase IA-encoding protein, and 12 phage- and prophage-related proteins. There were 31 proteins, which are only shared with some of the C. *sakazakii* genomes by C. *sakazakii* SP291 and which were absent among the other six species. These included a sodium-dependent phosphate transporter protein, a RelE antibacterial toxin protein, a RelB protein (antitoxin to RelE), a probable poly (beta-D-mannuronate) O-acetylase protein, two putative periplasmic proteins, a possible secretory protein, a GTPase protein, denoted as NgrB, a mobile element protein, a galactoside O-acetyltransferase protein, a mannose-6-phosphate isomerase, class I protein, a different locus type I restriction-modification system, specificity the S subunit-like protein, a predicted transcriptional regulator COGs COG2378, permeases of the major facilitator superfamily, a superfamily II DNA/RNA helicases, SNF2 family, a DNA modification methylase, an IS*1* transposase OrfA protein, a probable *tonB*-dependent receptor *yncD* precursor, a putative ORF-4 protein, a putative ORF (located using Glimmer/Genemark), seven beta-fimbriae probable major subunits, and four phage related proteins.

### Annotated plasmids contained in *C. sakazakii* SP291

**Figure A2:**
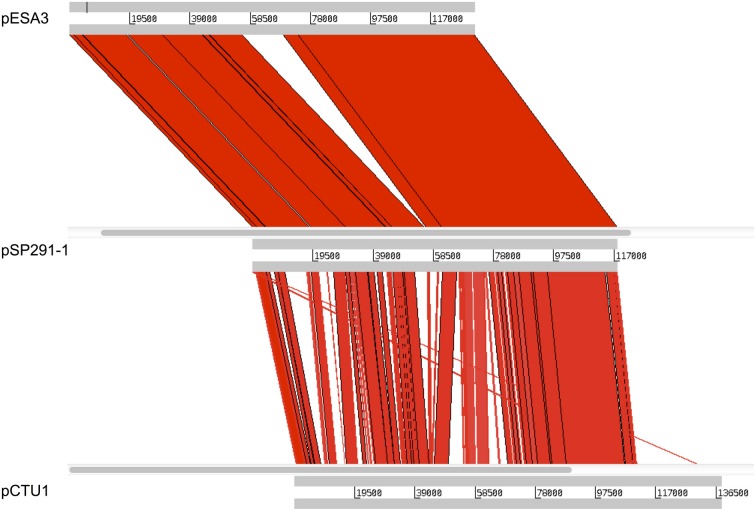
**Comparision of *plasmid group 1* (pESA3, pSP291-1, and pCTU1) using Artemis comparison tool (ACT)**.

**Figure A3:**
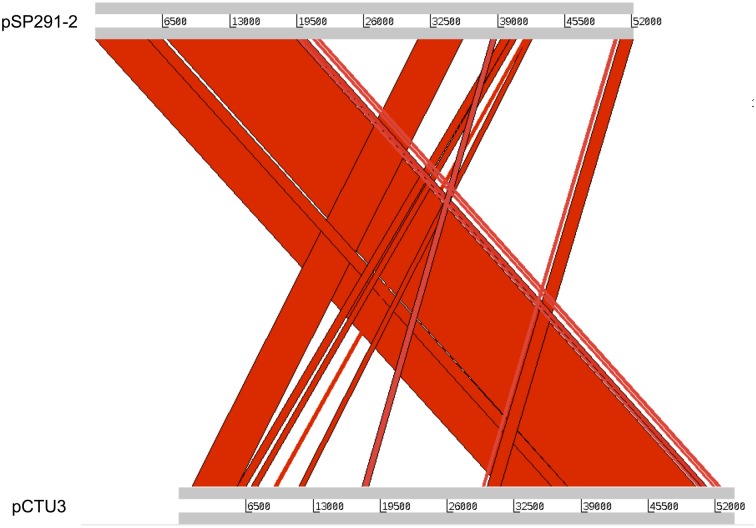
**Comparision of *plasmid group 2* (pSP291-2 and pCTU3) using Artemis comparison tool (ACT)**.

*Cronobacter sakazakii* SP291 contains three plasmids, including pSP291-1, 118,136 bp (57.2% GC), pSP291-2, 52,134 bp (49.2%) and pSP291-3, 4,422 bp (54.0% GC). The predicted CDS of pSP291-1 was found to be 116, with the average length of 887 bp, while pSP291-2 has 74 CDS with an average length of 544 bp, and pSP291-3 has seven CDS and with the average length of 307 bp (Table 2). Comparision of all three plasmids with five previously published plasmid sequences (including pESA2 and pESA3 of *C. sakazakii* ATCC BAA-894; along with pCTU1, pCTU2, and pCTU3 of *C. turicensis* z3032) indicated two closely related plasmid groups. *Plasmid group 1*, contains pSP291-1, pESA3, and pCTU1, while *plasmid group 2*, consists of pSP291-2 and pCTU3 (Figures A2, A3, and Table S5).

Several common genes were identified in *plasmid group 1*, These consisted of a complete ABC transporter (which could function to transport iron; vitamin B12; siderophores and hemin), including the ATP-binding component, the periplasmic substrate-binding module and the permease element. These genes were identified in all three plasmids. An aerobactin siderophore receptor (the IutA/TonB-dependent siderophore receptor) was shared between the three plasmids, while a *Cronobacter* plasminogen activator (*cpa*) homolog has only been mapped to pESA3 and pSP291-1, but not pCTU1, which is in agreement with the results reported by Franco et al. (2011) and Grim et al. (2012) (Figure A2). Three arsenical resistance genes were identified on all three plasmids along with pCTU3. Genes corresponding to commonly shared proteins on all three plasmids included a C-terminal helicase protein, a HipA protein previously reported to be required for growth arrest and multi-drug resistance in *Escherichia coli* (Correia et al., 2006), a hypothetical-encoding gene *ycgF* reported to be a direct anti-repressor which acts in the blue-light response of *E. coli* (Tschowri et al., 2009), a starvation sensing protein RspA, a magnesium transporting P-type 1 ATPase protein, transcriptional regulators, including members of ArsR family, GntR family (Kucerova et al., 2010; Joseph et al., 2012), LysR family and TetR-family, a MFS superfamily transporter, a Trk system encoding the potassium uptake protein TrkG, and an uncharacterized protein ImpD. A two-component response regulator protein, a two-component system sensor protein, three uncharacterized proteins ImpB, ImpC, and ImpJ/VasE, a glutathione S-transferase protein, a membrane protein, suppressor for copper-sensitivity ScsB, a hypothetical ABC transport system, periplasmic component, a RND efflux transporter, a suppression of copper sensitivity: putative copper binding protein ScsA were shared between pSP291-1 and pESA3, but not pCTU1, which confirmed the findings from previous studies (Kucerova et al., 2010; Joseph et al., 2012). In *plasmid group 2*, 15 heavy metal (copper, cobalt, zinc, cadmium, lead, and mercury) resistance genes were shared by both plasmids (Figure A3). An osmosensitive K^+^ channel histidine kinase protein (KdpD), and a virulence-associated protein *vagC* were also present in both plasmids. PCR analysis confirmed the presence of a pCTU3 IncH1-like origin of replication gene, *repA* in *C. sakazakii* SP291 (data not shown).

Interestingly, pSP291-1 contained two unique proteins, a histone acetyltransferase HPA2 and related acetyltransferases protein, along with an uncharacterized protein ImpH/VasB. Six specific proteins were found in pSP291-2, which included a putative glutathione S-transferase protein, a LysR family transcriptional regulator, a putative phage-associated acyl carrier protein, a S-adenosylmethionine: tRNA ribosyltransferase-isomerase protein, permeases of the major facilitator superfamily and an abortive infection protein. Various pSP291-3 proteins including mobilization proteins MobB, MobC, MobD, and DNA relaxase MbeA, which were not shared with any of the other plasmids, were also identified.

### Comparative phenotypic profiling of *C. sakazakii* ATCC BAA-894 and SP291

**Figure 2:**
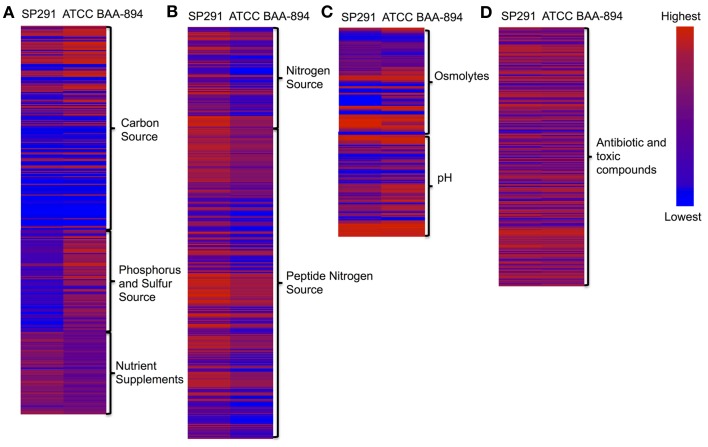
**(A–D)** Heat map of phenotype microarray (PM). Left lane, *C. sakazakii* SP291; Right lane, *C. sakazakii* ATCC BAA-894.

The phenotypic microarray (PM) platform was used previously to support the re-classification of this bacterial genus (Iversen et al., 2008). By comparing the phenotypes of *C. sakazakii* ATCC BAA-894 and *C. sakazakii* SP291 expressed across the complete array, interesting differences were observed and these were subsequently assessed in light of comparisons made at the genome level despite of the same PIF orgin. General differences, noted in the phenotypes between the two strains were described in the form of a heat map shown in Figure 2 (the corresponding numerical data is shown in Table S6). Phenotypic differences based on the bacteria's ability to utilize carbon, nitrogen, phosphorous and sulfur sources, as well as other nutrient supplements were noted. Furthermore, growth responses to osmolytes and different pH growth environments, as measured by the array were also observed for both strains. Antibiotic resistance patterns and the ability to respire in the presence of toxic compounds differed.

#### Carbon, nitrogen, phosphorous, and sulfur, other nutrient supplement metabolite utilization

Bacteria require a sufficient supply of various biomolecules to support their metabolic activity. In natural environments, where these bacteria are often found, it is to be expected that only limited amounts of these nutrients may be available. To support efficient adaptation and to enable growth in these conditions, bacteria will evolve redundant metabolic systems to support the utilization of a broad range of different substrates, with varying efficiencies. The PM array data gives an insight into how these features differ, between *C. sakazakii* SP291 and *C. sakazakii* ATCC BAA-894.

A number of phenotypic differences based on their ability to utilize a range of carbon sources were noted (Figure 2A and Table S6). When compared with *C. sakazakii* ATCC BAA-894, *C. sakazakii* SP291 could grow faster in m-inositol and slower in succinic acid, dulcitol, D,L-α-glycerol phosphate, D,L-malic acid, Tween 20, α-ketoglutaric acid, uridine, bromosuccinic acid, glycolic acid, inosine, and dextrin. In contrast there were little or no differences in growth rates when other carbon sources such as methyl pyruvate, mannose, and β-methyl-D-glucuronic acid were compared.

Differences in phenotypes based on the metabolism of carbon sources were compared at the genome level within the carbohydrate subsystem (Table S7). Interestingly, nine inositol catabolism genes were annotated in the *C. sakazakii* SP291 genome (Table S8), which supported the PM data. Furthermore, a pentose phosphate pathway gene, a lactose utilization gene, and a sucrose utilization gene were also annotated in the *C. sakazakii* SP291 genome specifically although no evidence to support their activity was found following PM analysis. Similarly, a maltose and maltodextrin utilization gene and a lactate utilization gene were annotated in *C. sakazakii* ATCC BAA-894 alone, with supporting evidence for the activity lacking from the PM array data. In all, 428 annotated genes related to carbon metabolism were shared between *C. sakazakii* ATCC BAA-894 and *C. sakazakii* SP291, which included 10 chitin and N-acetylglucosamine utilization genes, five fructoselysine (amadori product) utilization pathway genes, five dehydrogenase complexes genes, a dihydroxyacetone kinases gene, 14 Entner-Doudoroff pathway genes, and others.

Dancer et al. (2009a,b) reported that for *Cronobacter* species the availability and utilization of a nitrogen source was an important determinant for biofilm formation when growing in skim milk, and that strong biofilm formers were responsible for coagulation of skim milk (Dancer et al., 2009a). Data from the phenotypic microarray, showed no differences in nitrogen metabolism when *C. sakazakii* ATCC BAA-894 and *C. sakazakii* SP291 were compared (Figure 2B and Table S6). Interestingly, when regions of these two genomes known to encode genes associated with nitrogen metabolism were compared, a 16-kb locus, consisting of eight genes was found to be absent in *C. sakazakii* SP291 compared to *C. sakazakii* ATCC BAA-894 (Table 3). BLAST analysis of the region facilitated the identification of the corresponding genes located at this position. This locus, contained two nitrate transport proteins NrtB and NrtC, two nitrite reductase proteins NasB and NasA, a respiratory nitrate reductase NarL, a nitrate/nitrite-sensing protein NarX, a nitrite extrusion protein 1 NarK, and a nitrate reductase 1, alpha subunit NarG. This region was also present in *C. sakazakii* ES15 and *C. turicensis* z3032. Furthermore, 24 nitrate genes were broadly shared between both of the genomes, which was supported by data from the PM analysis.

**Table 3:** **Genes associated with nitrogen metabolism, comparing *C. sakazakii* SP291 and *C. sakazakii* ATCC BAA 894**.

**Function**	**Gene**	**Size (bp)**	***C. sakazakii* SP291**	***C. sakazakii* ATCC BAA-894**
Nitrite-sensitive transcriptional repressor NsrR	*nsrR*	426	166,807…167,232	171,773…172,198
Nitrogen regulatory protein P-II	*glnB*	339	729,723…730,061	676,465…676,803
Flavohemoprotein (Hemoglobin-like protein) (Flavohemoglobin) (Nitric oxide dioxygenase)	*hmp*	1191	731,488…730,298	677,084…678,274
Nitrate/nitrite response regulator protein	*narP*	645	809,507…810,151	756,306…756,950
Nitrogen assimilation regulatory protein Nac	*nac*	918	1,231,697…1,232,614	1,195,036…1,195,953
Response regulator NasT	*nasT*	1203	1,528,174…1,529,376	1,450,050…1,451,252
Nitrate ABC transporter, nitrate-binding protein	*nrtA*	1251	1,530,111…1,531,361	1,451,992…1,453,242
Nitrate ABC transporter, permease protein	*nrtB*	881	Absent	1,453,252…1,454,133
Nitrate ABC transporter, ATP-binding protein	*nrtC*	788	Absent	1,454,143…1,454,931
Nitrite reductase [NAD(P)H] large subunit	*nasB*	4067	Absent	1,454,941…1,459,008
Assimilatory nitrate reductase large subunit	*nasA*	2702	Absent	1,459,005…1,461,707
Nitrate/nitrite response regulator protein	*narL*	651	Absent	1,463,744…1,464,394
Nitrate/nitrite sensor protein	*narX*	1809	Absent	1,464,387…1,466,195
Nitrite extrusion protein 1	*narK*	1407	Absent	1,466,493…1,467,899
Respiratory nitrate reductase alpha chain	*narG*	3747	Absent	1,468,317…1,472,063
Respiratory nitrate reductase beta chain	*narH*	1311	1,531,371…1,532,681	1,472,060…1,473,595
Respiratory nitrate reductase delta chain	*narJ*	711	1,532,678…1,533,388	1,473,592…1,474,302
Respiratory nitrate reductase gamma chain	*narI*	678	1,533,388…1,534,065	1,474,302…1,474,979
Oxygen-insensitive NAD(P)H nitroreductase/Dihydropteridine reductase	*nfnB*	645	1,851,923…1,852,567	1,807,325…1,807,969
ABC-type nitrate/sulfonate/bicarbonate transport systems, periplasmic components		1161	1,856,747…1,857,907	1,812,312…1,813,310
Fumarate and nitrate reduction regulatory protein	*fnr*	753	1,932,047…1,932,799	1,887,404… 1,888,156
Hydroxylamine reductase	*hcp*	1653	2,402,562…2,404,214	2,413,065…2,414,717
NADH oxidoreductase hcr	*hcr*	969	2,404,225…2,405,193	2,414,728… 2,415,696
Oxygen-insensitive NADPH nitroreductase	*nfsA*	723	2,428,664…2,429,386	2,434,920…2,434,198
Nitrilotriacetate monooxygenase component B		618	2,510,602…2,511,219	2,487,772…2,488,389
Nitrogen regulatory protein P-II	*glnK*	339	2,833,540…2,833,878	2,792,459…2,792,797
PTS system nitrogen-specific IIA component, PtsN	*ptsN*	534	3,536,000…3,536,533	3,531,047…3,531,580
Phosphocarrier protein, nitrogen regulation associated	*ptsO*	273	3,537,449…3,537,721	3,532,496…3,532,768
Nitrogen regulation protein NR(I)	*glnG*	1410	4,009,029…4,010,438	3,993,113…3,994,522
Nitrogen regulation protein NR(II)	*glnL*	1050	4,010,447…4,011,496	3,994,531…3,995,580
Nitrite reductase [NAD(P)H] small subunit	*nirD*	327	4,309,983…4,310,309	4,334,267…4,334,593
Nitrite reductase [NAD(P)H] large subunit	*nirB*	2547	4,310,306…4,312,852	4,334,590…4,337,136

*C. sakazakii* SP291 was found to grow significantly slower in minimal media supplemented with phosphorous containing compounds (Figure 2A and Table S6), particularly in O-phospho-D-tyrosine, phospho-L-arginine, D,L-α-glycerol phosphate, β-glycerol phosphate, phosphoryl choline, phosphoenol pyruvate, D-glucose-6-phosphate, adenosine 3′-monophosphate, guanosine 2′-monophosphate, guanosine 3′-monophosphate, guanosine 5′-monophosphate, guanosine 2′,3′-cyclic monophosphate, cytidine 2′-monophosphate, cytidine 3′-monophosphate, thymidine 5′-monophosphate, and uridine 5′-monophosphate. Genome annotation provided a conflicting view as determined by the genes identified (Table S9). Twenty-nine phosphorus metabolism genes were broadly shared between *C. sakazakii* SP291 and *C. sakazakii* ATCC BAA-894, including eight high affinity phosphate transporters and control of *pho*-related regulon genes, 18 phosphate metabolism genes, and three polyphosphate genes. *Cronobacter* species cultured from a PIF production site were compared for their ability to grow in different food matrices (Cooney, 2012). Some demonstrated a slower growth rate compared to others, a feature that might contribute to their enhanced survival in this environment.

No differences in the metabolism of sulfur containing compounds were observed following a comparison of these strains after PM analysis (Figure 2A and Table S6). Forty-nine sulfur metabolism genes were shared by *C. sakazakii* ATCC BAA-894 and *C. sakazakii* SP291 (Table S10). These consisted of 17 inorganic sulfur assimilation genes, eight alkanesulfonate assimilation genes, five alkanesulfonates utilization genes, six L-cystine uptake and metabolism genes, four taurine utilization genes, three galactosylceramide and sulfatide metabolism genes, and six thioredoxin-disulfide reductase genes.

Iron is an essential nutrient for bacterial growth and the process of iron acquisition is generally thought to be a prerequisite for a pathogen to establish an infection when entering a host, a feature previously reported in *Cronobacter* species (Crosa and Walsh, 2002; Franco et al., 2011; Grim et al., 2012). High-affinity iron binding molecules, such as siderophores, and specific iron transport systems function to sequester iron from the environment when bacteria are subjected to iron-limiting growth conditions (Grim et al., 2012). Interestingly, analysis of the PM data showed no major differences between *C. sakazakii* SP291 and *C. sakazakii* ATCC BAA-894, in terms of their metabolism of iron or other nutrient supplements. Several transport systems were annotated in *C. sakazakii* SP291, and which are shared with *C. sakazakii* ATCC BAA-894 (Kucerova et al., 2010; Joseph et al., 2012), including a ferric hydroxamate ABC transporter denoted as FhuCDBA, 16 ferric enterobactin transporter proteins (including EntA, EntE, EntD, EntB, Fes, EntS, EntF, YbdZ, FepC, FepD, FepG, FepE, FepB, EntC, FepA2, and EntH), a ferrous iron transporter EfeUOB, along with a hemin transporter system, including a ferric reductase protein FhuF and a periplasmic binding protein TonB. A gene summary of iron acquisition and metabolism markers in *C. sakazakii* SP291 chromosome is shown in Table S11.

Additionally, iron acquisition and metabolism genes were also identified on pSP291-1 (Table S5), which were indistinguishable from that previously reported to be present on pESA3 of *C. sakazakii* ATCC BAA-894 (Kucerova et al., 2010; Joseph et al., 2012) and pCTU1 of *C. turicensis* z3032 (Franco et al., 2011; Grim et al., 2012). Target genes from previous reports, such as the RepFIB-like origin of replication gene *repA*, two plasmid-borne iron acquisition systems (*eitCBAD* and *iucABCD/iutA*), as well as the *Cronobacter* plasminogen activator *cpa* gene were all present in pSP291-1, with no evidence of the 17-kb type VI secretion system (T6SS) locus identified previously in pESA3 along with a 27-kb region encoding a filamentous hemagglutinin gene (*fhaB*), its specifc transporter gene (*fhaC*), and associated putative adhesins (FHA locus) identified in pCTU1 (Kucerova et al., 2010; Franco et al., 2011; Grim et al., 2012, 2013; Joseph et al., 2012). These features support the hypothesis that these plasmids have evolved from a single archetypal backbone that included an iron acquisition system. Our sequence analysis and those of other groups (Joseph et al., 2012; Grim et al., 2013) did not find evidence of plasmid mobilization genes associated with the several *plasmid group 1* genomes.

#### Osmolyte tolerance and survival in different pH environments

When present in different environments, bacteria must develop strategies that promote their survival. Genetic adaptation is derived from modifications of gene expression, *via* mutations, the acquisition of new and beneficial gene traits, or when these new traits are brought under control of a regulator that was already present in the core genome of the organism's ancestor (Maurelli, 2007). The outcome is that the organism is now better equipped to survive within the new ecological niche. It is generally thought that genes that are no longer compatible with the new lifestyle are selectively inactivated either by point mutation, insertion, or deletion and the contribution of gene loss to an organism's evolution is only now beginning to be appreciated (Maurelli, 2007). Based on our understanding of *Cronobacter* species epidemiology, these organisms are considered as environmental bacteria. Therefore their ability to survive adverse conditions would be critical. Phenotypes associated with growth in a range of osmolytes and in different pH growth environments were measured by PM analysis (Figure 2C and Table S6) as an indirect reflection of challenging environmental niches. In response to the presence of osmolytes, *C. sakazakii* SP291 could tolerate 100 mM sodium nitrate compared with *C. sakazakii* ATCC BAA-894. In contrast, the former grew slower in solutions containing 5% NaCl, 4% potassium chloride, 4% urea, 4–11% sodium lactate, 200 mM sodium phosphate at pH 7 and 20 mM sodium benzoate at pH 5.2. These observations are consistent with what has been suggested previously, in that when a selected adaptation event occurs, and the bacterium enters a new environment such as the human host, phenotypes change (Tall, unpublished observations). Comparing the ability of the environmental isolate *C. sakazakii* SP291 to survive over a range of different pH growth conditions with that of the PIF isolate *C. sakazakii* ATCC BAA-894, the former grew faster in a growth condition of pH 9.5 with phenylethylamine, whilst its growth was slower in pH 4.5 with L-proline. This example demonstrates the gain of one phenotype consistent with the inability to survive in the human host (ability to survive in high pH growth conditions) compared to the loss of a sufficient acid resistance response. In this case, the pathoadaptative event that resulted in an increased persistence in the environment comes at the expense of decreased commensal fitness of the microbe (a patent acid response) to survive the acidity of the stomach. However, a greater number of genomes and strains should be evaluated to rule out strain to strain variation.

Annotation of the genome suggested that *C. sakazakii* SP291 contained a repertoire of genes that could function to aid survival under stressed conditions, such as osmolyte tolerance and different pH environments (Table 4). One hundred and fifty-two annotated genes were identified as being involved with various stress responses. Their presence in the genome may provide early insights into how *C. sakazakii* SP291 adapts to and survives under different stressful growth conditions.

**Table 4:** **A selection of the stress response-encoding genes, the defined sub-system, together with the gene name, length of the ORF and correspondoing function, identified in *C. sakazakii* SP291**.

**Category**	**Sub-system**	**Gene**	**Size (bp)**	**Function**
Osmotic stress	Choline and betaine uptake and betaine biosynthesis	*betB*	1472	Betaine aldehyde dehydrogenase
Osmotic stress	Choline and betaine uptake and betaine biosynthesis	*betA*	1679	Choline dehydrogenase
Osmotic stress	Choline and betaine uptake and betaine biosynthesis	*betI*	608	HTH-type transcriptional regulator BetI
Osmotic stress	Choline and betaine uptake and betaine biosynthesis	*betT*	2030	High-affinity choline uptake protein BetT
Osmotic stress	Choline and betaine uptake and betaine biosynthesis	*opuCA*	1145	Glycine betaine/carnitine/choline transport ATP-binding protein OpuCA
Osmotic stress	Choline and betaine uptake and betaine biosynthesis	*opuCB*	647	Glycine betaine/carnitine/choline transport ATP-binding protein OpuCB
Osmotic stress	Choline and betaine uptake and betaine biosynthesis	*opuCC*	905	Glycine betaine/carnitine/choline transport ATP-binding protein OpuCC
Osmotic stress	Choline and betaine uptake and betaine biosynthesis	*opuCD*	713	Glycine betaine/carnitine/choline transport ATP-binding protein OpuCD
Osmotic stress	Choline and betaine uptake and betaine biosynthesis	*proP*	1506	L-Proline/Glycine betaine transporter ProP
Osmotic stress	Choline and betaine uptake and betaine biosynthesis	*proV*	1202	L-Proline/Glycine betaine ABC transport system permease protein ProV
Osmotic stress	Choline and betaine uptake and betaine biosynthesis	*proW*	1070	L-proline glycine betaine ABC transport system permease protein ProW
Osmotic stress	Choline and betaine uptake and betaine biosynthesis	*proX*	995	L-proline glycine betaine binding ABC transporter protein ProX
Osmotic stress	Osmoprotectant ABC transporter YehZYXW of enterobacteriales	*yehX*	941	Osmoprotectant ABC transporter ATP-binding subunit YehX
Osmotic stress	Osmoprotectant ABC transporter YehZYXW of enterobacteriales	*yehZ*	908	Osmoprotectant ABC transporter binding protein YehZ
Osmotic stress	Osmoprotectant ABC transporter YehZYXW of enterobacteriales	*yehW*	731	Osmoprotectant ABC transporter inner membrane protein YehW
Osmotic stress	Osmoprotectant ABC transporter YehZYXW of enterobacteriales	*yehY*	1133	Osmoprotectant ABC transporter permease protein YehY
Osmotic stress	Osmoregulation	*aqpZ*	695	Aquaporin Z
Osmotic stress	Osmoregulation	*glpF*	848	Glycerol uptake facilitator protein
Osmotic stress	Osmoregulation	*osmY*	614	Osmotically inducible protein OsmY
Osmotic stress	Osmoregulation	*ompA*	1076	Outer membrane protein A precursor
Osmotic stress	Osmoregulation	*yiaD*	662	Inner membrane lipoprotein yiaD
Osmotic stress	Osmotic stress cluster	*yciM*	1169	Heat shock (predicted periplasmic) protein YciM, precursor
Osmotic stress	Osmotic stress cluster	*osmB*	215	Osmotically inducible lipoprotein B precursor
Osmotic stress	Osmotic stress cluster	*pgpB*	764	Phosphatidylglycerophosphatase B
Osmotic stress	Osmotic stress cluster	*yciT*	800	Transcriptional regulatory protein YciT
Osmotic stress	Synthesis of osmoregulated periplasmic glucans	*mdoH*	2528	Glucans biosynthesis glucosyltransferase H
Osmotic stress	Synthesis of osmoregulated periplasmic glucans	*mdoC*	1157	Glucans biosynthesis protein C
Osmotic stress	Synthesis of osmoregulated periplasmic glucans	*mdoD*	1715	Glucans biosynthesis protein D precursor
Osmotic stress	Synthesis of osmoregulated periplasmic glucans	*mdoG*	1553	Glucans biosynthesis protein G precursor
Osmotic stress	Synthesis of osmoregulated periplasmic glucans	*opgC*	1220	OpgC protein
Osmotic stress	Synthesis of osmoregulated periplasmic glucans	*mdoB*	2294	Phosphoglycerol transferase I
Cold shock	Cold shock, CspA family of proteins	*cspA*	212	Cold shock protein CspA
Cold shock	Cold shock, CspA family of proteins	*cspC*	209	Cold shock protein CspC
Cold shock	Cold shock, CspA family of proteins	*cspD*	230	Cold shock protein CspD
Cold shock	Cold shock, CspA family of proteins	*cspE*	209	Cold shock protein CspE
Cold shock	Cold shock, CspA family of proteins	*cspG*	212	Cold shock protein CspG
Heat shock	Heat shock dnaK gene cluster extended	*dnaJ*	1139	Chaperone protein DnaJ
Heat shock	Heat shock dnaK gene cluster extended	*dnaK*	1700	Chaperone protein DnaK
Heat shock	Heat shock dnaK gene cluster extended	*yggX*	275	FIG001341:,Probable Fe(2+)-trafficking protein YggX
Heat shock	Heat shock dnaK gene cluster extended	*gshB*	947	Glutathione synthetase
Heat shock	Heat shock dnaK gene cluster extended	*grpE*	602	Heat shock protein GrpE
Heat shock	Heat shock dnaK gene cluster extended	*rdgB*	593	Nucleoside 5-triphosphatase RdgB (dHAPTP, dITP, XTP-specific)
Heat shock	Heat shock dnaK gene cluster extended	*rpoH*	857	RNA polymerase sigma factor RpoH
Heat shock	Heat shock dnaK gene cluster extended	*hemN2*	1136	Radical SAM family enzyme, similar to coproporphyrinogen III oxidase, oxygen-independent, clustered with nucleoside-triphosphatase RdgB
Heat shock	Heat shock *dnaK* gene cluster extended	*rph*	635	Ribonuclease PH
Heat shock	Heat shock dnaK gene cluster extended	*rsmE*	731	16S rRNA methyltransferase RsmE
Heat shock	Heat shock dnaK gene cluster extended	*prmA*	881	Ribosomal protein L11 methyltransferase
Heat shock	Heat shock dnaK gene cluster extended	*hslR*	401	Ribosome-associated heat shock protein implicated in the recycling of the 50S subunit (S4 paralog)
Heat shock	Heat shock dnaK gene cluster extended	*lepA*	1799	Translation elongation factor LepA
Heat shock	Heat shock dnaK gene cluster extended	*yraL*	860	rRNA small subunit methyltransferase I
Heat shock	Heat shock dnaK gene cluster extended	*smpB*	482	tmRNA-binding protein SmpB
Dessication stress	O-antigen capsule important for environmental persistence	*yihT*	875	Aldolase YihT
Dessication stress	O-antigen capsule important for environmental persistence	*yihS*	1241	Aldose-ketose isomerase YihS
Dessication stress	O-antigen capsule important for environmental persistence	*yihQ*	2030	Alpha-glucosyltransferase YihQ
Dessication stress	O-antigen capsule important for environmental persistence	*yihW*	806	DeoR-type transcriptional regulator YihW
Dessication stress	O-antigen capsule important for environmental persistence	*yihO*	1430	Glucuronide transport protein YihO
Dessication stress	O-antigen capsule important for environmental persistence	*yihP*	1406	Glucuronide transport protein YihP, homologous to YihO
Dessication stress	O-antigen capsule important for environmental persistence	*yshA*	686	Outer membrane sugar transport protein YshA
Dessication stress	O-antigen capsule important for environmental persistence	*yihU*	899	Oxidoreductase YihU
Dessication stress	O-antigen capsule important for environmental persistence	*yihV*	899	Sugar kinase YihV
Dessication stress	O-antigen capsule important for environmental persistence	*yihR*	866	Sugar-1-epimerase YihR
Detoxification	D-tyrosyl-tRNA(Tyr) deacylase	*dtd*	437	D-tyrosyl-tRNA(Tyr) deacylase
Detoxification	Glutathione-dependent pathway of formaldehyde detoxification	*frmA*	1118	S-(hydroxymethyl)glutathione dehydrogenase
Detoxification	Glutathione-dependent pathway of formaldehyde detoxification	*yieG*	830	S-formylglutathione hydrolase YeiG
Detoxification	Tellurite resistance: chromosomal determinants	*ydsK*	980	Uncharacterized acetyltransferase ydcK
Detoxification	Tellurite resistance: chromosomal determinants	*tehB*	593	Tellurite resistance protein TehB
Detoxification	Tellurite resistance: chromosomal determinants	*ydcL*	668	Uncharacterized lipoprotein ydcL
Detoxification	Uptake of selenate and selenite	*dedA*	659	DedA protein
Detoxification	Uptake of selenate and selenite	*cysA*	1094	Sulfate and thiosulfate import ATP-binding protein CysA
Detoxification	Uptake of selenate and selenite	*tsgA*	1187	TsgA protein homolog
Oxidative stress	Glutaredoxins	*yebA*	1331	Uncharacterized metalloprotease yebA
Oxidative stress	Glutaredoxins	*yibP*	1259	Uncharacterized protein yibP
Oxidative stress	Glutaredoxins	*hmp*	1190	Flavohemoprotein (Hemoglobin-like protein) (Flavohemoglobin) (Nitric oxide dioxygenase)
Oxidative stress	Glutaredoxins	*grxB*	647	Glutaredoxin 2
Oxidative stress	Glutaredoxins	*grxC*	251	Glutaredoxin 3 (Grx3)
Oxidative stress	Glutaredoxins	*nrdH*	245	Glutaredoxin-like protein NrdH, required for reduction of Ribonucleotide reductase class Ib
Oxidative stress	Glutaredoxins	*grlA*	347	Probable monothiol glutaredoxin GrlA
Oxidative stress	Glutathione: biosynthesis and gamma-glutamyl cycle	*ggt*	1766	Gamma-glutamyltranspeptidase
Oxidative stress	Glutathione: biosynthesis and gamma-glutamyl cycle	*gshA*	1556	Glutamate-cysteine ligase
Oxidative stress	Glutathione: biosynthesis and gamma-glutamyl cycle	*gshB*	947	Glutathione synthetase
Oxidative stress	Glutathione: non-redox reactions	*rnhA*	716	FIG005121: SAM-dependent methyltransferase
Oxidative stress	Glutathione: non-redox reactions	*gst1*	668	Glutathione S-transferase
Oxidative stress	Glutathione: non-redox reactions	*yghU*	866	Uncharacterized Glutathione S-transferase like protein yghU
Oxidative stress	Glutathione: non-redox reactions	*gst*	608	Glutathione S-transferase
Oxidative stress	Glutathione: non-redox reactions	*yqjG*	986	Uncharacterized protein yqjG
Oxidative stress	Glutathione: non-redox reactions	*gloB*	755	Hydroxyacylglutathione hydrolase
Oxidative stress	Glutathione: non-redox reactions	*gloA*	407	Lactoylglutathione lyase
Oxidative stress	Glutathione: non-redox reactions	*yfcF*	644	Probable glutathione S-transferase, YfcF homolog
Oxidative stress	Glutathione: non-redox reactions	*yfcG*	626	Probable glutathione S-transferase, YfcG homolog
Oxidative stress	Glutathione: non-redox reactions	*yibF*	608	Uncharacterized GST-like protein yibF
Oxidative stress	Glutathione: non-redox reactions	*yliJ*	626	Uncharacterized glutathione S-transferase-like protein
Oxidative stress	Glutathione: redox cycle	*grxB*	635	Glutaredoxin 2
Oxidative stress	Glutathione: redox cycle	*grxC*	251	Glutaredoxin 3 (Grx3)
Oxidative stress	Glutathione: redox cycle	*nrdH*	245	Glutaredoxin-like protein NrdH, required for reduction of Ribonucleotide reductase class Ib
Oxidative stress	Glutathione: redox cycle	*btuE*	551	Glutathione peroxidase
Oxidative stress	Glutathione: redox cycle	*lpd*	1427	Glutathione reductase
Oxidative stress	Glutathione: redox cycle	*gor*	1352	Glutathione reductase
Oxidative stress	Glutathionylspermidine and Trypanothione	*yjfC*	1187	Uncharacterized protein yjfC
Oxidative stress	Glutathionylspermidine and Trypanothione	*ygiC*	1160	Uncharacterized protein ygiC
Oxidative stress	NADPH:quinone oxidoreductase 2	*ytfG*	854	Uncharacterized oxidoreductase ytfG
Oxidative stress	NADPH:quinone oxidoreductase 2	*qorR*	380	Redox-sensing transcriptional regulator QorR
Oxidative stress	Oxidative stress	*katG*	2180	Catalase/peroxidase HPI
Oxidative stress	Oxidative stress	*katE*	2255	Hydroperoxidase II
Oxidative stress	Oxidative stress	*fur*	452	Ferric uptake regulation protein FUR
Oxidative stress	Oxidative stress	*dps*	503	DNA protection during starvation protein
Oxidative stress	Oxidative stress	*fnr*	752	Fumarate and nitrate reduction regulatory protein
Oxidative stress	Oxidative stress	*oxyR*	917	DNA-binding transcriptional regulator OxyR
Oxidative stress	Oxidative stress	*dps*	503	DNA protection during starvation protein
Oxidative stress	Oxidative stress	*sodA*	626	Manganese superoxide dismutase
Oxidative stress	Oxidative stress	*nsrR*	353	Nitrite-sensitive transcriptional repressor NsrR
Oxidative stress	Oxidative stress	*dpS*	503	Non-specific DNA-binding protein Dps
Oxidative stress	Oxidative stress	*osmC*	428	Organic hydroperoxide resistance
Oxidative stress	Oxidative stress	*ohrR*	548	Organic hydroperoxide resistance transcriptional regulator
Oxidative stress	Oxidative stress	*yebS*	1283	Inner membrane protein yebS
Oxidative stress	Oxidative stress	*pqiA*	1284	Paraquat-inducible protein A
Oxidative stress	Oxidative stress	*yebT*	2633	Uncharacterized protein yebT
Oxidative stress	Oxidative stress	*ymbA*	563	Uncharacterized lipoprotein ymbA
Oxidative stress	Oxidative stress	*pqiB*	1640	Paraquat-inducible protein B
Oxidative stress	Oxidative stress	*katG*	2180	Catalase/peroxidase HPI
Oxidative stress	Oxidative stress	*soxR*	458	Redox-sensitive transcriptional activator SoxR
Oxidative stress	Oxidative stress	*soxS*	323	Regulatory protein SoxS
Oxidative stress	Oxidative stress	*sodC*	518	Superoxide dismutase [Cu-Zn] precursor
Oxidative stress	Oxidative stress	*zur*	515	Zinc uptake regulation protein Zur
Oxidative stress	Protection from reactive oxygen species	*katG*	2180	Catalase/peroxidase HPI
Oxidative stress	Protection from reactive oxygen species	*katE*	2255	Hydroperoxidase II
Oxidative stress	Protection from reactive oxygen species	*sodA*	626	Manganese superoxide dismutase
Oxidative stress	Protection from reactive oxygen species	*sodC*	518	Superoxide dismutase [Cu-Zn] precursor
Oxidative stress	Redox-dependent regulation of nucleus processes	*gapA1*	995	NAD-dependent glyceraldehyde-3-phosphate dehydrogenase
Oxidative stress	Redox-dependent regulation of nucleus processes	*gapA2*	996	NAD-dependent glyceraldehyde-3-phosphate dehydrogenase
Oxidative stress	Redox-dependent regulation of nucleus processes	*npdA*	824	NAD-dependent protein deacetylase of SIR2 family
Oxidative stress	Redox-dependent regulation of nucleus processes	*pncA*	641	Nicotinamidase
Oxidative stress	Redox-dependent regulation of nucleus processes	*pncB*	1202	Nicotinate phosphoribosyltransferase
Periplasmic stress	Periplasmic stress response	*htrA*	1427	HtrA protease/chaperone protein
Periplasmic Stress	Periplasmic stress response	*skp*	494	Outer membrane protein H precursor
Periplasmic Stress	Periplasmic stress response	*degQ*	1367	Outer membrane stress sensor protease DegQ, serine protease
Periplasmic Stress	Periplasmic stress response	*degS*	1067	Outer membrane stress sensor protease DegS
Periplasmic Stress	Periplasmic stress response	*rseA*	650	Sigma factor RpoE negative regulatory protein RseA
Periplasmic Stress	Periplasmic stress response	*rseB*	854	Sigma factor RpoE negative regulatory protein RseB precursor
Periplasmic Stress	Periplasmic Stress response	*surA*	1286	Survival protein SurA precursor (Peptidyl-prolyl *cis-trans* isomerase SurA)
No subcategory	Bacterial hemoglobins	*hmp*	1190	Flavohemoprotein (Hemoglobin-like protein) (Flavohemoglobin) (Nitric oxide dioxygenase)
No subcategory	Carbon starvation		2105	Carbon starvation protein A
No subcategory	Carbon starvation	*cstA*	2153	Carbon starvation protein A paralog
No subcategory	Carbon starvation	*csrA*	185	Carbon storage regulator
No subcategory	Carbon starvation		584	Starvation lipoprotein Slp paralog
No subcategory	Carbon starvation	*rspA*	1316	Starvation sensing protein RspA
No subcategory	Carbon starvation	*sspA*	641	Stringent starvation protein A
No subcategory	Carbon starvation	*sspB*	491	Stringent starvation protein B
No subcategory	Commensurate regulon activation	*marA*	374	Multiple antibiotic resistance protein MarA
No subcategory	Commensurate regulon activation	*gpmB*	869	Probable phosphoglycerate mutase gpmB
No subcategory	Commensurate regulon activation	*soxS*	324	Regulatory protein SoxS
No subcategory	Commensurate regulon activation	*ramA*	344	Transcriptional activator RamA
No subcategory	Flavohaemoglobin	*hmp*	1190	Flavohemoprotein (Hemoglobin-like protein) (Flavohemoglobin) (Nitric oxide dioxygenase)
No subcategory	Hfl operon	*hflX*	1280	GTP-binding protein HflX
No subcategory	Hfl operon	*hflC*	1004	HflC protein
No subcategory	Hfl operon	*hflK*	1244	HflK protein
No subcategory	Hfl operon	*yjeT*	197	Putative inner membrane protein YjeT (clustered with HflC)
No subcategory	Hfl operon	*hfq*	308	RNA-binding protein Hfq
No subcategory	Phage shock protein (psp) operon	*pspA*	671	Phage shock protein A
No subcategory	Phage shock protein (psp) operon	*pspB*	224	Phage shock protein B
No subcategory	Phage shock protein (psp) operon	*pspC*	356	Phage shock protein C
No subcategory	Phage shock protein (psp) operon	*pspD*	242	Phage shock protein D
No subcategory	Phage shock protein (psp) operon	*pspF*	1001	Psp operon transcriptional activator
No subcategory	Sugar-phosphate stress regulation	*sgrR*	1664	SgrR, sugar-phosphate stress, transcriptional activator of SgrS small RNA
No subcategory	Universal stress protein family	*uspA*	437	Universal stress protein A
No subcategory	Universal stress protein family	*uspB*	335	Universal stress protein B
No subcategory	Universal stress protein family	*uspC*	422	Universal stress protein C
No subcategory	Universal stress protein family	*uspE*	956	Universal stress protein E
No subcategory	Universal stress protein family	*uspG*	428	Universal stress protein G

In recent studies involving *Salmonella* species, a picture of the transcriptome in low-moisture growth conditions has begun to emerge (Frossard et al., 2012; Finn et al., 2013). Allied to this, 25 genes involved in osmotic stress, and covering 16.4% of the stress response genes were identified in *C. sakazakii* SP291. Interestingly, the osmoprotectant ABC transporter denoted as YehZYXW in the *Cronobacter* genome, together with the L-proline glycine betaine MFS transporter ProP, and the ABC transporter ProU systems (composed of ProV, ProW, ProX) designed in *Escherichia coli* (Checroun and Gutierrez, 2004) and *Salmonella* Typhimurium (Cairney et al., 1985) were identified in the *C. sakazakii* SP291 genome. Moreover, an osmoregulator transporter including genes *opuCA*, *opuCB*, *opuCC*, and a fourth gene, which was also an ABC transporter denoted as *opuCD* here, was 77% similar to that of the *osmU* operon (*osmVWXY*) in *Salmonella* (Frossard et al., 2012) at the gene level. Other osmotically functioning genes identified included the betaine/carnitine/choline transporter (BCCT) family, which acts to transport betaine and choline. This operon consists of a high-affinity choline uptake gene *betT*, a helix-turn-helix (HTH)-type transcriptional regulator *betI*, which was previously identified in *E. coli* (Lamark et al., 1991), a betaine aldehyde dehydrogenase *betB* gene and a choline dehydrogenase gene *betA*. An *in silico* assessment of those loci involved in osomotolerance comparing *Cronobacter sakazakii* ATCC BAA-894 and *E. coli* K12 MG1655 identified these latter features also (Feeney and Sleator, 2011). Interestingly, several other genes linked to osmotic stress conditions were identified in the genome of *C. sakazakii* SP291, which included five osmoregulation genes (*aqpZ*, *glpF*, *osmY*, *ompA*, and *yiaD*), four osmotic stress cluster genes (*yciM*, *osmB*, *pgpB*, and *yciT*) and six osmoregulated periplasmic glucan genes (*mdoH*, *mdoC*, *mdoD*, *mdoG*, *opgC* and *mdoB*). None of these genes were identified previously by Feeney and Sleator (2011). Finally, *ompA* which encodes an outer membrane porin, was identified in *C. sakazakii* SP291 and is a recognized virulence marker (Kim et al., 2010).

Experiments to investigate the nature of the *C. sakazakii* responses to cold- and heat-shock conditions have been reported (Shaker et al., 2008; Carranza et al., 2010; Chang et al., 2010; Al-Nabulsia et al., 2011; Gajdosova et al., 2011). Following exposure to extreme temperatures of cold-shock at −20°C, or heat-shock at 47°C, survival of *Cronobacter sakazakii* was significantly enhanced (Chang et al., 2010). Carranza et al. (2010) reported that when exposed to higher temperatures, several potential virulence factors were up-regulated. The fact that the pathogenic potential of *Cronobacter* species may be related to its ability to survive at higher temperatures, warrents further investigation. From the genome sequence of *C. sakazakii* SP291, the *cspA* family of cold-shock genes (including *cspA, cspC, cspD, cspE* and *cspG*) along with 11 other genes annotated as heat-shock genes, part of the *dnaK* gene cluster, including *dnaJ, dnaK*, *yggX*, *gshB*, *grpE*, *rdgB*, *rpoH*, *hemN2*, *rph*, *rsmE*, *prmA*, *hslR*, *yraL*, and *smpB* genes were conserved.

**Figure 3:**
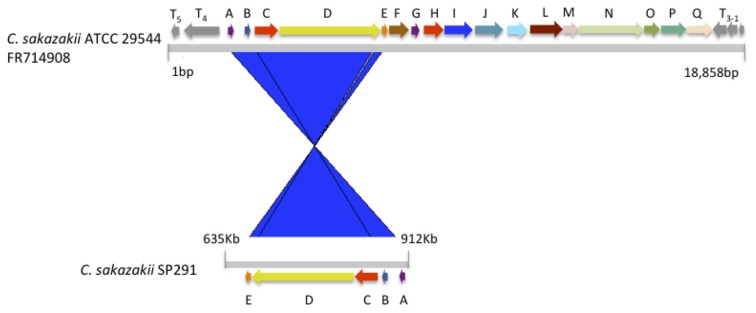
**The putative thermotolerance-containing DNA region of *C. sakazakii* ATCC 29544 compared with *C. sakazakii* SP291**.

Using a top-down proteomics approach, Williams et al. (2005) identified a candidate protein in *C. sakazakii*, known to be associated with thermotolerance in *Methylobacillus flagelatum*, and which was denoted as KT. In a recent study, the genomic region containing this presumptive marker of thermotolerance was compared to similar regions in other bacteria (Gajdosova et al., 2011). An *in silico* analysis showed that this thermotolerace KT-region was present in 4 of 14 isolates consisting of seven *Cronobacter* species studied by Joseph et al. (2012). *Cronobacter sakazakii* SP291 can survive desiccation for long periods of time at an average temperature of 56.7°C, similar to that recorded when spray drying is in operation during PIF production (Cooney, 2012). Interestingly, *C. sakazakii* SP291 was negative for the KT marker, as determined by PCR (data not shown). Apart from the locus between *orfA-orfE*, when the corresponding region of the SP291 genome was compared to that of *C. sakazakii* ATCC 29,544, this region was devoid of the KT-encoding homolog (Figure 3). In light of the thermo-adapted phenotype possessed by *C. sakazakii* SP291, this finding suggests that there may be other thermotolerance survival mechanisms expressed by *C. sakazakii* SP291.

As mentioned above, *Cronobacter* species have the capacity to survive in desiccated environments for long periods, a phenotype that is linked to their epidemiology and routes of infection. As an example of genes linked to this phenotype, the *yih*-encoding operons, consisted of 10 annotated genes present in the genome of *C. sakazakii* SP291. Desiccation-related proteins YihU, YihT, YihR, YihS, YihQ and YihV have conserved domains which function in carbohydrate transport and metabolism. YihO is a glucuronide transport protein whilst YihP is homologous to it. YshA is an outer membrane sugar transport protein and YihW is a *deoR*-type transcriptional regulator, reported to negatively regulate the expression of *yihU-oyshA* in *Salmonella* (Gibson et al., 2006). This operon was reported to be up-regulated following desscication stress in *Salmonella*. Interstingly, the *yih* operon are conserved in 17 annotated *Cronobacter* genomes (strain information of the genomes are listed in Table 1) and noted previously (Grim et al., 2013).

The ability of a bacterium to eliminate toxic compounds from the cell is an important survival mechanism. Nine genes involved in detoxification were identified in the *C. sakazakii* SP291 genome. These included a tellurite resistance-encoding gene *tehB*, which matches a 593 bp hypothetical protein in *C. sakazakii* ATCC BAA-894. However, a tellurite resistance region (*terACDYZ*) was reported only in *C. sakazakii* ATCC BAA-894 (Table 4), and with the exception of the *terC*-encoding marker in *C. turicensis* z3032, was not identified in any of the other *Cronobacter* species genomes sequenced (Kucerova et al., 2010; Joseph et al., 2012; Grim et al., 2013). Genes involved in the detoxification of organic pollutants, including a D-tyrosyl-tRNA (Tyr) deacylase-encoding *dtd*, two glutathione-dependent pathway formaldehyde detoxification genes (*frmA* and *yieG*), three genes involved in the uptake of selenate and selenite (*dedA, cysA*, and its homolog *tsgA*), and two uncharacterized genes (*ydsK* and *ydcL*), were also identified in *C. sakazakii* SP291. This feature supports an earlier report describing the ability of *Cronobacter* species to detoxify and survive in tannery wastewater effluents (Chandra et al., 2011).

Oxidative stress is an example of an important bacterial stress response, with 62 annotated genes covering 40.8% genome linked to this sub-system. These genes included the zinc uptake regulation *zur*, which was reported as involved in the oxidative stress response of *Streptomyces coelicolor* (Shin et al., 2007). Other stress-related genes included seven periplasmic stress related genes, a bacterial hemoglobin gene, seven genes involved in carbon starvation, four commensurate regulon activation genes, a flavohaemoglobin gene, five *hfl* operon genes, five phage shock protein (*psp*) operon genes, a sugar-phosphate stress regulation gene, and five universal stress protein family genes.

#### Resistance to antibiotics and toxic compounds

*C. sakazakii* was originally reported to be susceptible to a panel of 69 antimicrobial agents (Stock and Wiedemann, 2002). Subsequently, a tetracycline-resistant *C. sakazakii* cultured from a Chilean freshwater salmon farm (Miranda et al., 2003) was isolated, followed by a report of a trimethoprim and neomycin resistance isolate cultured from fresh domiati cheese (El-Sharoud et al., 2009). More recently, isolates resistant to cephalothin were recovered from dried food (Chon et al., 2012). The emergence of strains that have become resistant to antimicrobial compounds is of great concern to public health (Dumen, 2010; Yan et al., 2012).

Figure 2D shows a heat map comparing *C. sakazakii* SP291 and *C. sakazakii* ATCC BAA-894 and Table 5 provides a summary of the significant changes in PM redox measurements, after bacterial growth in microtitre wells containing a number of antimicrobial compounds as part of the phenotypic microarray. Compared to *C. sakazakii* ATCC BAA-894, *C. sakazakii* SP291 exhibited more activity in the presence of 5,7-dichloro-8-hydroxyquinoline, 5-nitro-2-furaldehyde semicarbazone, hexamminecobalt (III) chloride, poly-L-lysine, protamine sulfate, ornidazole, tobramycin, streptomycin, apramycin, iodonitro tetrazolium violet, amoxicilin, neomycin, and sisomicin; while exhibiting a reduced activity in the presence of phleomycin, ciprofloxacin, cinoxacin, dichlofluanid, tolylfluanid, guanidine hydrochloride, colistin, methyl viologen, sodium azide, guanazole, rifamycin SV, glycine hydroxamate, D,L-methionine hydroxamate, cefmetazole, and cloxacillin.

**Table 5:** **Comparison of the differential phenotypes expressed by *C. sakazakii* SP291 and *C. sakazakii* ATCC BAA-894 related to antimicrobial and toxic compounds as determined by phenotype microarray**.

**Test compound**	**Difference^a^**	**Mode of action**
**PHENOTYPES GAINED BY *C. sakazakii* SP291 RELATIVE TO *C. sakazakii* ATCC BAA-894-**		
Amoxicillin	20,446	Wall, lactam
Neomycin	47,681	Protein synthesis, 30S ribosomal subunit, aminoglycoside
Sisomicin	40,297	Protein synthesis, 30S ribosomal subunit, aminoglycoside
Tobramycin	33,297	Protein synthesis, 30S ribosomal subunit, aminoglycoside
Sodium arsenate	22,155	Toxic anion, PO4 analog
Sodium metaborate	65,231	Toxic anion
EDTA	89,134	Chelator, hydrophilic
5,7-Dichloro-8-hydroxyquinoline	21,638	Chelator, lipophilic
5-Nitro-2-furaldehyde semicarbazone	40,348	DNA damage, multiple sites, nitrofuran analog
Protamine sulfate	27,720	Membrane, non-specific binding
Streptomycin	40,297	Protein synthesis, 30S ribosomal subunit, aminoglycoside
Potassium tellurite	27,025	Toxic anion
Sodium tungstate	50,849	Toxic anion, molybdate analog
Poly-L-lysine	43,324	Membrane, detergent, cationic
Sodium m-arsenite	28,927	Toxic anion
Sodium periodate	47,820	Toxic anion, oxidizing agent
Antimony (III) chloride	35,457	Toxic cation
Iodonitro tetrazolium violet	22,427	Respiration
Hexamminecobalt (III) Chloride	30,202	DNA synthesis
Apramycin	43,717	Protein synthesis, 30S ribosomal subunit, aminoglycoside
Ornidazole	22,594	Protein glycosolation
**PHENOTYPES LOST BY *C. sakazakii* SP291 RELATIVE TO *C. sakazakii* ATCC BAA-894-**		
Cloxacillin	−20,939	Wall, lactam
Colistin	−35,638	Membrane, transport
Guanidine hydrochloride	−47,347	Membrane, chaotropic agent
Cefmetazole	−21,713	Wall, cephalosporin second generation
Phleomycin	−45,183	DNA damage, oxidative, ionizing ratiation
Methyl viologen	−81,565	Oxidizing agent
Sodium azide	−22,375	Respiration, uncoupler
Dichlofluanid	−42,205	Fungicide, phenylsulphamide
Cinoxacin	−18,744	DNA unwinding, gyrase (GN), topoisomerase (GP), quinolone
Rifamycin SV	−22,130	RNA polymerase
Glycine hydroxamate	−64,946	tRNA synthetase
D,L−Methionine hydroxamate	−38,317	tRNA synthetase
Sodium bromate	−27,123	Toxic anion
Guanazole	−33,164	Ribonucleotide DP reductase
Ciprofloxacin	−22,172	DNA unwinding, gyrase (GN), topoisomerase (GP), fluoroquinolone
Tolylfluanid	−18,317	Fungicide, phenylsulphamide

a Denotes the following: a positive number indicates faster growth in C. sakazakii SP291 compared to C. sakazakii ATCC BAA-894; a negative number indicates faster growth in C. sakazakii ATCC BAA-894 compared to C. sakazakii SP291.

Careful analysis of the PM data showed an interesting phenotype, related to bioactive and toxic anions. *C. sakazakii* SP291 survived significantly better in sodium metaborate, potassium tellurite, sodium m-arsenite, sodium tungstate, sodium periodate, sodium arsenate, and antimony (III) chloride compared with *C. sakazakii* ATCC BAA-894. In contrast the latter bacterium, exhibited a distinct phenotype in sodium bromate. These observations suggested that *C. sakazakii* SP291 elaborates a greater ability to counter the effects of a broader range of heavy metals, a characteristic that could be facilitate adaptation in powered infant formula manufacturing environments where metallic compositions such as quaternary containing disinfectants are used for decontamination. This resistance phenotype may be globally regulated as well. Together, this information may explain how this organism adapted to the manufacturing environment.

Based in part on these phenotypes, a total of 44 genes were shared between *C. sakazakii* ATCC BAA-894 and *C. sakazakii* SP291. These consisted of adaptation to D-cysteine related genes (include *yecC*, *yecS*, and *dcyD*), a β-lactamase-encoding *ampC* gene, three cobalt-zinc-cadmium resistance genes (including *feiF*, *zitB* and a MerR family transcriptional regulator), 11 copper homeostasis genes (include *cueO*, *yobA*, *copA*, *zntA*, *ccmF*, *ccmH*, *cutE*, *cutF*, *corC*, *cutA*, and a copper resistance protein D gene), a fosfomycin resistance gene *fosA*, a lysozyme inhibitor *mliC*, a tripartite multidrug resistance system found in Gram-negative bacteria, 11 multidrug resistance efflux pumps (including *macB*, *macA*, *acrA*, *acrE*, *norM*, *acrD*, *acrB*, *acrF*, *acrR*, *envR*, and *tolC*), four genes encoding resistance to fluoroquinolones (including *gyrA*, *gyrB*, *parC*, and *parE*), and a multidrug resistance cluster (consisting of *mdtB*, *mdtC*, *mdtD*, *mdtA*, *baeR*, and *baeS*). All of these genes mapped to the bacterial chromosome. In addition three arsenic resistance genes were identified on pSP291-1 (Table S5). This latter feature confirmed the previous report on the copper/silver resistance determinants in *Cronobacter* species (Kucerova et al., 2010; Sivamaruthi et al., 2011; Joseph and Forsythe, 2012; Joseph et al., 2012; Grim et al., 2013). In contrast, eight cobalt-zinc-cadmium resistance genes (include *cusA*, *cusC*, *cusF*, *czcA*, *czcB*, *cusS*, *cusR*, *and pcoS*) and three copper homeostasis genes (including *copG*, *pcoB* and *pcoA*) were unique to *C. sakazakii* ATCC BAA-894, of which *cusRCFBA*/*silRECBA* and *pcoABCDR* were indicated as two copper and silver resistance regions. The previous region was shared among *C. sakazakii*, *C. malonaticus*, and *C. turicensis*; while the latter region was shared among *C. sakazakii*, *C. malonaticus*, *C. turicensis* and *C. universalis* (Joseph et al., 2012) (Table 6).

**Table 6:** **Genes related to resistance to antibiotics and toxic compounds annotated in *C. sakazakii* SP291 and *C. sakazakii* ATCC BAA-894**.

**Subsystem**	**Start**	**Stop**	**Size (bp)**	**Gene**	**Role**
**RESISTANCE TO ANTIBIOTIC AND TOXIC COMPUNDS GENES SHARED BY *C. sakazakii* SP291 AND *C. sakazakii* ATCC BAA-894-**					
Adaptation to D-cystine	1,345,012	1,345,764	752	*yecC*	Cystine ABC transporter, ATP-binding protein
Adaptation to d-cystine	1,344,347	1,345,015	668	*yecS*	Cystine ABC transporter, permease protein
Adaptation to d-cystine	1,343,343	1,344,323	980	*dcyD*	D-cystine desulfhydrase
Beta-lactamase	1,853,746	1,852,619	1127	*ampC*	Beta-lactamase
Cobalt-zinc-cadmium resistance	4,087,380	4,088,282	902	*fieF*	Cobalt-zinc-cadmium resistance protein
Cobalt-zinc-cadmium resistance	733,136	733,498	362		Transcriptional regulator, MerR family
Cobalt-zinc-cadmium resistance	2,570,193	2,571,155	962	*zitB*	Zinc transporter ZitB
Copper homeostasis	2,643,555	2,645,219	1664	*cueO*	Blue copper oxidase CueO precursor
Copper homeostasis	1,433,730	1,434,104	374	*yobA*	Copper resistance protein C precursor
Copper homeostasis	1,434,109	1,434,978	869		Copper resistance protein D
Copper homeostasis	2,766,031	2,768,538	2507	*copA*	Copper-translocating P-type ATPase
Copper homeostasis	4,209,899	4,207,683	2216	*zntA*	Zinc/cadmium/mercury/lead-transporting ATPase
Copper homeostasis	924,321	926,270	1949	*ccmF*	Cytochrome c heme lyase subunit CcmF
Copper homeostasis	926,821	927,282	461	*ccmH*	Cytochrome c heme lyase subunit CcmH
Copper homeostasis: copper tolerance	2,651,936	2,653,477	1541	*cutE*	Copper homeostasis protein CutE
Copper homeostasis: copper tolerance	3,053,870	3,053,172	698	*cutF*	Copper homeostasis protein CutF precursor
Copper homeostasis: copper tolerance	2,651,056	2,651,931	875	*corC*	Magnesium and cobalt efflux protein CorC
Copper homeostasis: copper tolerance	130,149	129,802	347	*cutA*	Periplasmic divalent cation tolerance protein CutA
Fosfomycin resistance	1,712,546	1,712,959	413	*fosA*	Fosfomycin resistance protein FosA
Lysozyme inhibitors	1,973,845	1,973,522	323	*mliC*	Membrane-bound lysozyme inhibitor of c-type lysozyme
Multidrug resistance, tripartite systems found in gram negative bacteria	2,468,114	2,466,543	1517	*emrB2*	Inner membrane component of tripartite multidrug resistance system
Multidrug resistance, tripartite systems found in gram negative bacteria	2,469,268	2,468,111	1157		Membrane fusion component of tripartite multidrug resistance system
Multidrug resistance, tripartite systems found in gram negative bacteria	2,466,541	2,465,000	1541	*nodT*	Outer membrane component of tripartite multidrug resistance system
Multidrug resistance efflux pumps	2,397,113	2,395,170	1943	*macB*	Macrolide export ATP-binding/permease protein MacB
Multidrug resistance efflux pumps	2,398,222	2,397,110	1112	*macA*	Macrolide-specific efflux protein MacA
Multidrug resistance efflux pumps	2,799,127	2,800,332	1205	*acrA*	Membrane fusion protein of RND family multidrug efflux pump
Multidrug resistance efflux pumps	3,599,205	3,600,347	1142	*acrE*	Membrane fusion protein of RND family multidrug efflux pump
Multidrug resistance efflux pumps	1,999,315	2,000,688	1373	*norM*	Multi antimicrobial extrusion protein (Na(+)/drug antiporter), MATE family of MDR efflux pumps
Multidrug resistance efflux pumps	809,372	806,256	3116	*acrD*	Probable aminoglycoside efflux pump
Multidrug resistance efflux pumps	2,800,354	2,803,503	3149	*acrB*	Acriflavine resistance protein B
Multidrug resistance efflux pumps	3,600,357	3,603,476	3119	*acrF*	Acriflavine resistance protein F
Multidrug resistance efflux pumps	2,798,982	2,798,317	665	*acrR*	HTH-type transcriptional regulator acrR
Multidrug resistance efflux pumps	3,598,827	3,598,180	647	*envR*	Transcription repressor of multidrug efflux pump acrAB operon, TetR (AcrR) family
Multidrug resistance efflux pumps	374,805	373,318	1487	*tolC*	Type I secretion outer membrane protein, TolC precursor
Resistance to fluoroquinolones	1,021,753	1,024,389	2636	*gyrA*	DNA gyrase subunit A
Resistance to fluoroquinolones	3,931,538	3,929,124	2414	*gyrB*	DNA gyrase subunit B
Resistance to fluoroquinolones	383,132	385,402	2270	*parC*	Topoisomerase IV subunit A
Resistance to fluoroquinolones	377,546	379,438	1892	*parE*	Topoisomerase IV subunit B
The mdtABCD multidrug resistance cluster	1,153,120	1,149,998	3122	*mdtB*	Multidrug transporter MdtB
The mdtABCD multidrug resistance cluster	1,149,997	1,146,908	3089	*mdtC*	Multidrug transporter MdtC
The mdtABCD multidrug resistance cluster	1,146,904	1,145,489	1415	*mdtD*	Multidrug transporter MdtD
The mdtABCD multidrug resistance cluster	1,154,367	1,153,120	1247	*mdtA*	Probable RND efflux membrane fusion protein
The mdtABCD multidrug resistance cluster	1,144,071	1,143,349	722	*baeR*	Response regulator BaeR
The mdtABCD multidrug resistance cluster	1,145,492	1,144,068	1424	*baeS*	Sensory histidine kinase BaeS
**RESISTANCE TO ANTIBIOTIC AND TOXIC COMPUNDS GENES SPECIFIC TO *C. sakazakii* ATCC BAA-894-**					
Cobalt-zinc-cadmium resistance	4,206,746	4,209,892	3146	*cusA*	Cation efflux system protein CusA
Cobalt-zinc-cadmium resistance	4,203,563	4,204,948	1385	*cusC*	Cation efflux system protein CusC precursor
Cobalt-zinc-cadmium resistance	4,204,976	4,205,329	353	*cusF*	Cation efflux system protein CusF precursor
Cobalt-zinc-cadmium resistance	4,206,746	4,209,892	3146	*czcA*	Cobalt-zinc-cadmium resistance protein CzcA
Cobalt-zinc-cadmium resistance	4,205,443	4,206,735	1292	*czcB*	Cobalt/zinc/cadmium efflux RND transporter, membrane fusion protein, CzcB family
Cobalt-zinc-cadmium resistance	4,202,505	4,201,219	1286	*cusS*	Copper sensory histidine kinase CusS
Cobalt-zinc-cadmium resistance	4,203,373	4,202,693	680	*cusR*	Copper-sensing two-component system response regulator CusR
Cobalt-zinc-cadmium resistance	4,219,774	4,221,174	1400	*pcoS*	Heavy metal sensor histidine kinase
Copper homeostasis	4,209,979	4,210,419	440	*CopG*	CopG protein
Copper homeostasis	4,217,266	4,217,688	422	*pcoB*	Copper resistance protein B
Copper homeostasis	4,214,975	4,216,792	1817	*pcoA*	Multicopper oxidase

## Conclusions

Stress responses and resistance to antibiotic and toxic compounds are interesting phenotypes identified in *C. sakazakii* SP291, when compared to *C. sakazakii* ATCC BAA-894, based on the comparative phenotypic microarray analysis in parallel with the annotation of its genome. Given this the fact that the PIF production environment is a stressful ecological niche, the osmoprotectant ABC transporters including YehZYXW, ProP, ProU, and OpuCABCD can be expected to play a role to support bacterial survival, as reported in other microorganisms previously (Cairney et al., 1985; Checroun and Gutierrez, 2004; Frossard et al., 2012; Finn et al., 2013). Notably, *C. sakazakii* SP291 possesses a greater ability to survive in a broader range of heavy metals, as quaternary containing disinfectants which include metallic compositions are often used for PIF manufacturing environment disinfection. In conclusion, genome analysis of *C. sakazakii* SP291 along with its metabolome highlighted a number of potential features, which might be considered as candidates for future studies to extend our understanding of the persistence and virulence mechanisms deployed by this bacterium in the production environment. These data provide an early insight into how a factory isolate may survive in a desiccation condition and adapt to the PIF production environment and associated food matrices.

### Conflict of interest statement

The authors declare that the research was conducted in the absence of any commercial or financial relationships that could be construed as a potential conflict of interest.

## Supplementary material

Table S1 **Genomic regions missing in *C. sakazakii* SP291 when compared to the other three completed genomes of *C. sakazakii* ATCC BAA-894 (as the reference sequence), *C. sakazakii* E15 and *C. turicensis* z3032**.

Table S2 **Genomic regions present only in *C. sakazakii* SP291 (as the reference sequence) when compared to the other three completed genomes of *C. sakazakii* ATCC BAA-894, *C. sakazakii* ES15 and *C. turicensis* z3032**.

Table S3 **Genome comparison of *C. sakazakii* SP291 with other available *C. sakazakii* genomes, which include *C. sakazakii* SP291, ATCC BAA-894, ES15, E899, 680, 696, 701, ES35, 2151, ES713, and E764**.

Table S4 **Genome comparison of *C. sakazakii* SP291 with *Cronobacter* isolate of other six species, which consist of *C. dublinensis* LMG 23823, *C. malonaticus* LMG 23826, *C. muytjensii* ATCC 51329, *C. turicensis* z3032, *C. universalis* NCTC 9529, and *C. condimenti* 1330**.

Table S5 **Annotated plasmid clusters of three complete genome, which include pSP291-1, pSP291-2, and pSP291-3 of *C. sakazakii* SP291, pESA2 and pESA3 of *C. sakazakii* ATCC BAA-894, as well as pCTU1, pCTU2, and pCTU3 of *C. turicensis* z3032. *Plasmid group 1*: pSP291-1, pESA3, and pCTU1; *plasmid group 2*: pSP291-2 and pCTU3**.

Table S6 **Numerical data for all 20 PM plates of *C. sakazakii* SP291 and *C. sakazakii* ATCC BAA-894, along with the phenotype differences (greater than 20,000±1,800 unit) generated by Omnilog® software**.

Table S7 **Genes assigned to defined bacterial sub-systems and their distribution among these functional categories for *C. sakazakii* SP291 along with other three completed genome, *C. sakazakii* ATCC BAA-894, *C. sakazakii* ES15, and *C. turicensis* z3032**.

Table S8 **Genome annotation: Carbohydrate metabolism of *C. sakazakii* SP291 and *C. sakazakii* ATCC BAA-894**.

Table S9 **Genome annotation: Phosphorus metabolism of *C. sakazakii* SP291 and *C. sakazakii* ATCC BAA-894**.

Table S10 **Genome annotation: Sulfur metabolism of *C. sakazakii* SP291 and *C. sakazakii* ATCC BAA-894**.

Table S11 **Iron acquisition systems identified in the chromosome of *C. sakazakii* SP291**.
